# Cultural adaptation and validation of the mandarin version of the scale of oral health outcomes for 5-year-old children

**DOI:** 10.3389/froh.2026.1749692

**Published:** 2026-01-29

**Authors:** Daijing Yu, Dawei Huang, Junhao Ai, Xiaoli Ma, Yan Hao, Qiuhua Pan, Sherry Shiqian Gao

**Affiliations:** 1 Department of Stomatology, School of Medicine, Xiamen University, Xiamen, China; 2 Department of Stomatology, Women and Children’s Hospital, School of Medicine, Xiamen University, Xiamen, China

**Keywords:** dental caries, oral health-related quality of life, preschool children, questionnaire, reliability, validity

## Abstract

**Introduction:**

To translate and culturally adapt the scale of oral health outcomes for 5-year-old children (SOHO-5) into Mandarin, and to assess the reliability and validity of the Mandarin version of the SOHO-5 (M-SOHO-5) for Mainland Chinese preschool children.

**Methods:**

A forward-backwards translation method was used to develop a draft M-SOHO-5 from the original English version. The draft was tested with 20 pairs of children and parents for the initial validity of the content, and revisions were made accordingly. Then, the final version was studied on 259 pairs of 5-year-old children and their parents. The reliability of M-SOHO-5 was assessed through test-retest reliability and internal consistency. Clinical examination for dental caries was performed to study the discriminant validity of M-SOHO-5. Construct validity was evaluated using global rating questions.

**Results:**

The Cronbach's alpha coefficients for the children's version of M-SOHO-5 (M-SOHO-5c) and the parents’ version (M-SOHO-5p) were 0.80 and 0.85, respectively. The intraclass correlation for M-SOHO-5c and M-SOHO-5p was 0.51 (*p* = 0.014) and 0.65 (*p* = 0.001). Children with dental caries had higher mean ranks for both M-SOHO-5c and M-SOHO-5p compared to caries-free children (130.88 vs. 124.68, *p* = 0.465; 136.58 vs. 114.72, *p* = 0.007). Moreover, the results of both M-SOHO-5c and M-SOHO-5p were correlated with the global rating questions.

**Discussion:**

The M-SOHO-5 showed good reliability and construct validity. The discriminant validity of the parental version is good, but that of the children's version is limited.

## 1 Introduction

Fédération Dentaire Internationale (FDI) World Dental Federation defines oral health as “it is multi-faceted and includes the ability to speak, smile, smell, taste, touch, chew, swallow and convey a range of emotions through facial expressions with confidence and without pain, discomfort and disease of the craniofacial complex (head, face, and oral cavity)” (1). This definition indicates that oral health is not merely the absence of disease, but a complete physical, psychological and social well-being of an individual. In clinical settings, dental professionals usually focus on oral disease diagnosis and have developed various objective measurements, such as using the decayed, missing, and filled teeth index to assess caries conditions and the community periodontal index to assess periodontal diseases (2, 3). However, little emphasis has been placed on patients' subjective perceptions of their oral condition. In light of this, dental researchers introduced the concept of Oral Health-Related Quality of Life (OHRQoL) in the early 2000s (4). OHRQoL is defined as “a multidimensional construct that reflects people's comfort when eating, sleeping and engaging in social interaction; their self-esteem; and their satisfaction with respect to their oral health” (5). This concept has been used to evaluate and understand people's feelings towards their oral condition in different circumstances.

In response to various oral diseases and functional needs, researchers have designed several OHRQoL diagnostic scales to meet the evaluation needs under different circumstances, allowing adult patients to express their subjective feelings. However, there are limited tools developed to measure the OHRQoL of children (6). Because preschool children are in the early stages of growth and development, their cognition, social psychology, and linguistic abilities are not sufficient to support them in accurately and fully expressing their emotions and thoughts (7). Therefore, the evaluation of OHRQoL of young children has always been challenging in oral healthcare (8). There are a few OHRQoL diagnostic scales available for use in young children at present, but the vast majority of these scales are completed by their parents (6). Those scales usually express what the parents believe to be their children's thoughts, rather than directly reflecting the true feelings of the children themselves.

In 2012, scholars developed a Scale of Oral Health Outcomes for 5-year-old children (SOHO-5) (8). It is the first structured questionnaire used to measure the OHRQoL of preschool children by both children's (SOHO-5c) and parental reports (SOHO-5p). SOHO-5c contains seven items, which refer to whether the child has any difficulties in eating, drinking, speaking, playing and sleeping because of his/her teeth, and avoids smiling due to pain or appearance. The answers are recorded by a 3-point scale: 0 = “No”, 1 = “A little”, and 2 = “A lot”. The total score of SOHO-5c is the sum of the scores of individual questions. The SOHO-5p also contains seven items, which refer to whether the parents consider their child has any difficulties in eating, speaking, playing and sleeping because of the child's teeth, avoids smiling due to pain or appearance, and whether their child's self-confidence is affected because of his/her teeth. The answers are recorded by a 5-point scale: 0 = “Not at all”, 1 = “A little”, 2 = “Moderate”, 3 = “A lot”, and 4 = “A great deal”. The total score of SOHO-5p is the sum of the scores of these seven questions. For both SOHO-5c and SOHO-5p, a higher score indicates a greater negative impact on the child and therefore a poorer OHRQoL of the child. Dental healthcare professionals can use these questions in an interview format to ask children about their feelings about their teeth; children can express their thoughts using verbal and visual methods. SOHO-5 has been translated and adapted by scholars from various countries and regions, including Bangladesh, Brazil, the Dominican Republic, Indonesia, Iran, Myanmar and Hong Kong (using the Cantonese dialect), for diagnosing the self-perceived OHRQoL of local preschool children (9–14). Studies from different regions indicate that SOHO-5 has good psychometric properties and is a reliable self-rated OHRQoL diagnostic scale for preschool children. However, there is no Mandarin Chinese version of SOHO-5, and it has not been used in preschool populations in mainland China. Therefore, the aim of this study is to translate and culturally adapt SOHO-5 into Mandarin Chinese and to assess the reliability and validity of the Mandarin version of the SOHO-5 (hereinafter referred to as M-SOHO-5) for preschool children in Xiamen, a city in the southeastern part of China.

## 2 Methods

This study was conducted in Xiamen, Fujian province, China, from November 2023 to December 2024. Ethical approval was obtained from the Institutional Review Board of the Medical Ethics Committee at Xiamen University (No. XDYX202305K29).

### 2.1 Cross-cultural adaptation

The Test Translation and Adaptation Guidelines of the International Test Commission were referred to for the cross-cultural adaptation of M-SOHO-5 in this study (15). A forward-backwards translation method was used to translate the original English version of SOHO-5 into Mandarin Chinese. First, the English SOHO-5c and SOHO-5p were independently translated into Mandarin by two bilingual translators; these two versions were compared and discussed in the research group to form a first draft of M-SOHO-5. Second, another two independent bilingual translators who were blinded to the original instrument translated the first draft of M-SOHO-5 back into English. The expression, semantics and concepts between the back-translated English version and the original SOHO-5 were checked. If there was any inconsistency between the two English versions, the first draft of M-SOHO-5 was modified accordingly to develop a second draft. The second draft of M-SOHO-5 was pre-tested on 20 pairs of 5-year-old children and their parents in one local kindergarten. The team members recorded the difficulties and obstacles encountered by the children in understanding the content of the questionnaire during the interview. The members also collected the opinions and suggestions of the participating parents regarding the language expression used in the second draft. After sorting out and summarising the relevant feedback, further modification was performed to finalise the questionnaires. The final version of M-SOHO-5 includes both the children's version (hereinafter referred to as M-SOHO-5c) and the parental version (hereinafter referred to as M-SOHO-5p).

### 2.2 Calculation of sample size and recruitment of participants

In this study, the sample size was calculated based on internal conformance tests (Cronbach's alpha statistics). M-SOHO-5 has seven questions for both the children's version and the parental version. The Cronbach's alpha value for each version is set to be 0.9, and the probability of type Ⅰ error is 0.05. Therefore, this study required the participation of at least 221 pairs of 5-year-old children and their parents (16). A sample size of 221 was calculated using Bonett's precision-based method to achieve a 95% confidence interval of approximate width 0.04 for an expected Cronbach's *α* = 0.9 in a 7-item scale. This sample size can also achieve a 95% confidence interval of approximate width 0.08 for an expected Cronbach's *α* = 0.8 (0.76–0.84) or approximate width 0.12 for an expected Cronbach's *α* = 0.7 (0.64–0.76) in a 7-item scale.

Three kindergartens in Xiamen were invited to participate in this study. The inclusion criteria were children 1) who were 5 years old, 2) who were able to cooperate with dental examinations, 3) who had no serious systemic diseases, and 4) who could understand and speak Mandarin. The exclusion criteria were children 1) who were under special dental treatments (such as orthodontic treatments), and 2) whose parents could not read the parental version of the questionnaire. A parental informed consent form was sent to all the children who met the inclusion criteria. Written consent was obtained before the study.

### 2.3 Questionnaire survey

M-SOHO-5c was completed before the dental check-up. A one-on-one interview was performed by three trained interviewers (DY, XM and JA) in the classroom of the kindergarten. Each child was asked to answer all the questions in M-SOHO-5c, including seven questions regarding their dental health-related quality of life and two additional global rating questions to assess the construct validity. The two global rating questions are 1) “How happy are you with your teeth?”, and 2) “Do you have any holes in your teeth?”. Duplicate interviews were performed two weeks later for randomly chosen 10% of the participating children by the same interviewers using the same questionnaire.

M-SOHO-5p was distributed to the parents of participating children in advance and collected before the dental check-up. Parents were asked to answer all the questions in M-SOHO-5p, including seven questions regarding their children's dental health-related quality of life and four additional global rating questions to assess the construct validity. The four global rating questions are 1) “Overall, how would you rate your child's dental health?”, 2) “Overall, how happy are you with your child's dental health?”, 3) “Do you think your child needs any dental treatment because of the state (holes in teeth, pain) of his/her teeth?”, and 4) “Do you think the well-being of your child is affected by the conditions of their teeth, lips, jaws or mouth?”. Duplicate questionnaires were sent and collected two weeks later for randomly chosen 10% of the participating parents.

### 2.4 Clinical examination

The clinical examination of dental caries status was performed in the kindergarten by one experienced dentist (DH). The clinical procedure referred to the Oral health surveys: basic methods - 5th edition, published by the World Health Organization (17). Children were asked to rinse their mouths before the examination. The dentist used a 0.5-mm ball-ended Community Periodontal Index probe to diagnose dental caries in children. The caries status was recorded using the dmft index, which refers to decayed, missing (due to caries) and filled primary teeth. Repeated examinations were conducted on randomly chosen 10% of the participating children to measure the intra-examiner reliability.

### 2.5 Statistical analysis

Two project assistants independently entered the data into a computer Excel sheet. The two input results were compared to check whether there were errors in data entry. Kappa statistics were used to evaluate intra-examiner reliability. The reliability of M-SOHO-5 was evaluated through measures of test-retest reliability and internal consistency. Test-retest reliability was measured by the degree of agreement between the answers to the first and duplicate questionnaires using intra-class correlation coefficients (ICCs). Internal consistency was determined using item-total correlation, the overall Cronbach's alpha coefficient, and Cronbach's alpha coefficient if the item is deleted. The validity of M-SOHO-5 was measured by discriminant validity and construct validity. Discriminant validity was assessed by the association between M-SOHO-5 scores and the dental caries status of participant children using the Mann–Whitney U-test. Construct validity was assessed by the Spearman correlation coefficient of the association between M-SOHO-5 scores and the results of the global rating questions. All the data analysis was performed using Statistical Package for the Social Sciences (SPSS) version 25.0 software (IBM, United States of America). The statistical significance of all tests (p-value) was set to 0.05.

## 3 Results

We invited 363 5-year-old children and their parents to participate in this study, and 345 children-parent pairs (95%) accepted the invitation. Children who were absent from school on the examination day and who could not cooperate with all the procedures were excluded from this study. Therefore, 259 children and their parents (75%) were included in this study. The prevalence of dental caries was 70% among the participating children, with a mean dmft index of 3.61 (SD = 3.92). About one-third (34%) of the children reported that their dental condition had at least one adverse effect on their oral health-related quality of life. The mean M-SOHO-5c score was 0.78 (SD = 1.77), ranging from 0 to 12. In addition, 28% of parents reported that their children have experienced negative consequences in daily activities because of the children's dental problems. The mean M-SOHO-5p score was 0.79 (SD = 2.10), with a range between 0 and 18 (Table 1). Detailed distribution of the responses for each question of M-SOHO-5c and M-SOHO-5p was presented in Table 2 and Table 3, respectively.

**Table 1:** Descriptive data of caries experience, M-SOHO-5c score and M-SOHO-5p score.

Item	Prevalence (higher than 0)	Mean ± SD	Minimum number	Maximum number
dmft	70%	3.61 ± 3.92	0	18
M-SOHO-5c score	34%	0.78 ± 1.77	0	12
M-SOHO-5p score	28%	0.79 ± 2.10	0	18

M-SOHO-5c, Mandarin children's self-report of the scale of oral health outcomes for 5-year-old children; M-SOHO-5p, Mandarin parental report of the scale of oral health outcomes for 5-year-old children; SD, standard deviation; dmft, decayed, missing (due to caries) and filled teeth.

**Table 2:** Distribution of the responses of M-SOHO-5c.

Item	Response (number, prevalence)		
	No	A little	A lot
Difficulty in eating	223, 86%	30, 12%	6, 2%
Difficulty in drinking	249, 96%	8, 3%	2, 1%
Difficulty in speaking	239, 92%	19, 7%	1, 1%
Difficulty in playing	237, 92%	15, 6%	6, 2%
Avoid smiling due to pain	233, 90%	21, 8%	5, 2%
Avoid smiling due to appearance	229, 89%	17, 7%	11, 4%
Difficulty in sleeping	237, 91%	15, 6%	7, 3%

M-SOHO-5c, Mandarin children's self-report of the scale of oral health outcomes for 5-year-old children.

**Table 3:** Distribution of the responses of M-SOHO-5p.

Item	Response (number, prevalence)				
	Not at all	A little	Moderate	A lot	A great deal
Difficulty in eating	204, 79%	36, 14%	17, 7%	1, 0%	1, 0%
Difficulty in speaking	244, 94%	11, 5%	3, 1%	1, 0%	0, 0%
Difficulty in playing	250, 97%	6, 2%	3, 1%	0, 0%	0, 0%
Avoid smiling due to appearance	243, 94%	9, 4%	6, 2%	1, 0%	0, 0%
Avoid smiling due to pain	245, 94%	9, 4%	4, 2%	1, 0%	0, 0%
Difficulty in sleeping	238, 92%	17, 7%	3, 1%	0, 0%	1, 0%
Influence self-confidence	240, 92%	15, 6%	2, 1%	2, 1%	0, 0%

M-SOHO-5p, Mandarin parental report of the scale of oral health outcomes for 5-year-old children.

The overall Cronbach's alpha value of M-SOHO-5c was 0.80 (Table 4). Deleting any item in M-SOHO-5c will result in an equivalent or decrease in its Cronbach's alpha value. Corrected item-total correlation for all the items was equal to or higher than 0.40, which was considered a moderately strong relationship (18). The overall Cronbach's alpha value of M-SOHO-5p was 0.85 (Table 5). However, if the item “difficulty in eating because of his/her teeth” was deleted, the Cronbach's alpha value of M-SOHO-5p could be increased to 0.89. The corrected item-total correlation ranged from 0.45 to 0.83, indicating a good relationship between a single item and the whole questionnaire. A total of 52 children (20%) participated in the retest of M-SOHO-5c. The ICCs of M-SOHO-5c were 0.51 [95% confidence interval (CI) was 0.07 to 0.74, p-value = 0.014]. Meanwhile, 49 parents (19%) completed the retest questionnaire of M-SOHO-5p. The results demonstrated that M-SOHO-5p had a medium test-retest reliability (ICCs = 0.65, *p* = 0.001) (Table 4 and Table 5).

**Table 4:** Reliability analysis of M-SOHO-5c.

Item	Internal consistency reliability			Test-retest reliability	
	Cronbach's alpha	Cronbach's alpha if item deleted	Corrected item-total correlation	Interclass correlation coefficients (95% CI)	*p*-value
Total score	0.80	–	–	0.51 (0.07-0.74)	0.014
Difficulty in eating	–	0.79	0.46	–	–
Difficulty in drinking	–	0.80	0.40	–	–
Difficulty in speaking	–	0.78	0.49	–	–
Difficulty in playing	–	0.77	0.57	–	–
Avoid smiling due to pain	–	0.76	0.60	–	–
Avoid smiling due to appearance	–	0.76	0.61	–	–
Difficulty in sleeping	–	0.76	0.63	–	–

M-SOHO-5c, Mandarin children's self-report of the scale of oral health outcomes for 5-year-old children; CI, confidence interval.

**Table 5:** Reliability analysis of M-SOHO-5p.

Item	Internal consistency reliability			Test-retest reliability	
	Cronbach's alpha	Cronbach's alpha if item deleted	Corrected item-total correlation	Interclass correlation coefficients (95% CI)	*p*-value
Total score	0.85	–	–	0.65 (0.34–0.82)	0.001
Difficulty in eating	–	0.89	0.45	–	–
Difficulty in speaking	–	0.82	0.74	–	–
Difficulty in playing	–	0.82	0.83	–	–
Avoid smiling due to appearance	–	0.84	0.58	–	–
Avoid smiling due to pain	–	0.82	0.73	–	–
Difficulty in sleeping	–	0.83	0.65	–	–
Influence self-confidence	–	0.82	0.70	–	–

M-SOHO-5p, Mandarin parental report of the scale of oral health outcomes for 5-year-old children; CI, confidence interval.

The results of the discriminant validity were presented in Table 6. Although children with dental caries showed a higher mean rank of the total score of M-SOHO-5c than children without dental caries, the result was not significant (130.88 vs. 124.68, *p* = 0.465). On the other hand, children with dental caries showed a significantly higher mean rank of the total score of M-SOHO-5p than children without dental caries (136.58 vs. 114.72, *p* = 0.007), which indicated that M-SOHO-5p had a satisfactory discriminant validity. The total scores of both versions of M-SOHO-5 were significantly associated with most of the global rating questions (Table 7 and Table 8). Children with higher M-SOHO-5c scores were less satisfied with their oral health (r = −0.194, *p* = 0.002). An increase in M-SOHO-5p score was correlated with lower parents-rated oral health of their children (r = −0.421, *p* < 0.001), less satisfaction with their children's teeth (r = −0.459, *p* < 0.001), higher parental perceived treatment need for their children (r = 0.362, *p* < 0.001), and more parental reported negative impact on children's general health (r = 0.532, *p* < 0.001).

**Table 6:** Discriminant validity of M-SOHO-5.

Item	Mean rank		
	Caries free	Caries	*p*-value
M-SOHO-5c total score	124.68	130.88	0.465
M-SOHO-5p total score	114.72	136.58	0.007

M-SOHO-5c, Mandarin children's self-report of the scale of oral health outcomes for 5-year-old children; M-SOHO-5p, Mandarin parental report of the scale of oral health outcomes for 5-year-old children.

**Table 7:** Construct validity of M-SOHO-5c.

Item	Satisfaction		Self-reported caries	
	*r*	*p*-value	*r*	*p*-value
Total score	−0.194	0.002	0.121	0.064
Difficulty in eating	−0.119	0.057	0.177	0.007
Difficulty in drinking	−0.042	0.497	0.068	0.302
Difficulty in speaking	−0.132	0.034	0.132	0.043
Difficulty in playing	−0.154	0.013	0.107	0.103
Avoid smiling because of toothache	−0.217	<0.001	0.061	0.354
Avoid smiling because of appearance	−0.157	0.012	0.247	<0.001
Difficulty in sleeping	−0.170	0.006	0.055	0.398

M-SOHO-5c, Mandarin children's self-report of the scale of oral health outcomes for 5-year-old children.

**Table 8:** Construct validity of C-SOHO-5p.

Item	Parents-rated oral health		Satisfaction		Treatment need		Impact on general health	
	*r*	*p*-value	*r*	*p*-value	*r*	*p*-value	*r*	*p*-value
Total score	−0.421	<0.001	−0.459	<0.001	0.362	<0.001	0.532	<0.001
Difficulty in eating	−0.364	<0.001	−0.379	<0.001	0.301	<0.001	0.311	<0.001
Difficulty in speaking	−0.187	0.002	−0.218	<0.001	0.212	0.001	0.415	<0.001
Difficulty in playing	−0.147	0.018	−0.141	0.023	0.136	0.042	0.345	<0.001
Avoid smile because of appearance	−0.145	0.020	−0.177	0.004	0.072	0.282	0.425	<0.001
Avoid smile because of toothache	−0.167	0.007	−0.180	0.004	0.147	0.028	0.437	<0.001
Difficulty in sleeping	−0.188	0.002	−0.170	0.006	0.201	0.002	0.382	<0.001
Influence self-confidence	−0.180	0.004	−0.196	0.002	0.165	0.013	0.472	<0.001

M-SOHO-5p, Mandarin parental report of the scale of oral health outcomes for 5-year-old children.

## 4 Discussion

This study successfully translated and validated SOHO-5 in Chinese Mandarin and supported its use in 5-year-old children and their parents in Xiamen, Fujian province, China. It is the first tool in the Mandarin language for preschool children to self-rate their OHRQoL. All the questions in the original SOHO-5 were retained in the Mandarin version, and M-SOHO-5 shows good reliability and acceptable validity. Moreover, all 5-year-old children who participated in the interview were able to understand and answer the questions of M-SOHO-5c smoothly, laying the foundation for promoting the use of this scale by dental healthcare workers in Fujian Province and other regions of China to diagnose OHRQoL in children.

The results of this study showed that the mean scores of M-SOHO-5c and M-SOHO-5p were relatively low (less than 1) despite the high caries prevalence in this group of children. This finding is similar to the Cantonese version (13) but lower than other language versions (9, 11, 12, 14). There may be two major reasons. First, we only recorded caries experience in this study. Other oral health-related conditions, for example, pulp involvement, trauma experience or self-reported toothache, were not included due to the feasibility issue. Although caries prevalence was high in the study group, dental caries cannot completely represent the discomfort that the children felt because of their teeth. Future studies can be performed to understand the correlation between M-SOHO-5 and different oral conditions in more detail. Second, children tend to habituate to chronic pain as time passes (19), so the discomfort may not influence their daily life functions and quality of life (20). The results of this study showed some differences between children and their parents in reporting the OHRQoL of the children. In general, more children reported at least one daily life-related negative impact caused by their teeth than their parents. Children also expressed a higher concern that their teeth condition could influence speaking, playing and sleeping activities. In particular, more than 10% children reported that they avoided smiling due to the appearance of their teeth. However, only 6% parents reported this behaviour. These findings were consistent with the Cantonese version (13). The Cantonese version reported that more children reported negative impacts caused by their teeth than their parents did. They also found that the responses of “avoided smiling due to the appearance of their teeth” were significantly different between the children's version and the parents' version. These results indicated that children themselves might be more sensitive to their personal feelings about oral health. Therefore, oral health researchers should not only rely on parental reports to study OHRQoL in young children, but also need to fully consider the direct feedback from children themselves.

In this study, internal consistency was assessed by two aspects: 1) whether all the items in M-SOHO-5 measured the same concept, and 2) whether the items were closely related to each other as a whole (21). The overall Cronbach's alpha value of M-SOHO-5c was 0.80, which was higher than the results in the original study (Cronbach's alpha=0.74) (7) and similar to other language versions (Brazilian – 0.77, Cantonese – 0.71, Myanmar – 0.82) (9, 13, 14). For a scale containing only seven items, such a Cronbach's alpha value was considered quite high, as lower reliability was common in questionnaires with fewer items (22). If any item was deleted in M-SOHO-5c, the Cronbach's alpha value would remain the same or decrease. In addition, the corrected item-total correlation of all items was considered a moderately strong relationship between items (18). Therefore, all of this evidence indicates that M-SOHO-5c has favourable internal consistency. For M-SOHO-5p, although the Cronbach's alpha value slightly increased after removing the item “difficulty in eating because of his/her teeth”, the increase was very small (from 0.85 to 0.89). This situation was also identified in the Cantonese version and Myanmar version (13, 14). In addition, this item was strongly correlated with the prevalence of dental caries in children and all the global rating questions in M-SOHO-5p. Therefore, we decided to keep this significant question in the questionnaire. Except for this issue, M-SOHO-5p showed high Cronbach's alpha values and high item-total correlations, demonstrating its good internal consistency.

This study used ICCs to evaluate the test-retest reliability of the questionnaire, aiming to measure the correlation and consistency of the answers provided by the same participant at different time points (23). In this study, the ICCs values of both M-SOHO-5c and M-SOHO-5p were considered at a moderate level with statistical significance (24). Our ICCs values were lower than other language versions (Brazilian and Myanmar – higher than 0.9, Persian – 0.8, Indonesian – higher than 0.8) (9, 11, 12), while the Cantonese version reported relatively lower test-retest reliability for the parents' version (13). It is worth noting that a relatively low ICC value does not necessarily mean a low degree of consistency, which may also be due to a small number of participants in the test-retest or a lack of sufficient variability between answers (25). In this study, only 52 children and 49 parents joined in the retest, which may be one reason for the relatively low ICC values.

Dental caries status was used in all other language versions to study the discriminant validity (8–14). Given that dental caries was the most widespread oral problem among preschool children in Xiamen, this study used the presence of dental caries as the basis for evaluating the discriminant validity of the M-SOHO-5 (26). Most of the other versions demonstrated that the total score of SOHO-5 in caries-free children was significantly lower than that in children with caries experience (8–14). The results showed that children with dental caries had higher M-SOHO-5c and M-SOHO-5p scores than children without caries experience. However, the result of M-SOHO-5c was not significant. The main reason might be that the result of “avoid smiling due to pain in his/her teeth” was in contrast to other items. Because the Cronbach's alpha if this item were deleted was lower than the overall Cronbach's alpha value, and the corrected item-total correlation of this item was good, we decided to keep this item in M-SOHO-5c. Nevertheless, the interpretation of the results is important and should be paid more attention to. Moreover, it was worth noting that most of the single items in M-SOHO-5c and M-SOHO-5p did not show a significant correlation to caries status. Therefore, it is recommended to take M-SOHO-5 as a whole to evaluate the general OHRQoL in Mainland Chinese children. In addition, more extensive research on young child populations in other regions of China is needed to further validate the above findings.

This study adopted Spearman's correlation coefficient to evaluate the construct validity of M-SOHO-5 because the results of M-SOHO-5 did not follow normal distributions (27). The results showed a significant correlation between the scores of both M-SOHO-5c and M-SOHO-5p with the answers to most of the global questions, indicating that M-SOHO-5 has good construct validity. Our findings were consistent with other language versions (9–14). However, it was worth noting that the correlation between the total M-SOHO-5c score and children's perception of dental caries was considered statistically non-significant. The main reason for this result may be the insufficient sample size. In this study, the sample size was determined by Cronbach's alpha statistics because it is the primary parameter of a newly developed questionnaire (28). However, the sample size may not be sufficient for Spearman's correlation coefficient analysis. Further studies can consider slightly increasing the sample size to meet the requirements for other statistical analyses.

This study has some limitations. First, we used dental caries status as the only oral health-related condition to measure the discriminant validity for M-SOHO-5. Future studies should consider including pulpal status or the presence of dental trauma experience to evaluate the validity of M-SOHO-5 on different occasions. Second, this study is a small-scale local study. Future studies should be performed in other communities to achieve a larger sample size. Third, the discriminant validity of M-SOHO-5c in dental caries was not statistically significant. The solitary interpretation of M-SOHO-5c data should be taken with caution. Oral health workers should combine the opinions from both children and their parents to achieve a more comprehensive understanding of young children's OHRQoL.

## 5 Conclusion

The Mandarin version of SOHO-5 exhibits high internal consistency reliability, moderate test-retest reliability and discriminant validity, and good construct validity. This tool can be used to evaluate the OHRQoL of 5-year-old children in communities where Mandarin is the primary language.

## Data Availability

The raw data supporting the conclusions of this article will be made available by the authors, without undue reservation.
